# Patterns of Genetic Diversity and the Invasion of Commercial Starters in *Saccharomyces cerevisiae* Vineyard Populations of Santorini Island

**DOI:** 10.3390/foods9050561

**Published:** 2020-05-02

**Authors:** Ioanna Chalvantzi, Georgios Banilas, Chrysoula Tassou, Aspasia Nisiotou

**Affiliations:** 1 Institute of Technology of Agricultural Products, Hellenic Agricultural Organization “Demeter”, Sofokli Venizelou 1, GR-14123 Lykovryssi, Greece; ichalvantzi@uniwa.gr (I.C.); ctassou@nagref.gr (C.T.); 2 Department of Wine, Vine and Beverage Sciences, University of West Attica, Ag. Spyridonos 28, GR-12243 Athens, Greece; gban@uniwa.gr

**Keywords:** wine yeasts, genetic diversity, interdelta PCR, genotyping, *Saccharomyces cerevisiae*

## Abstract

Autochthonous *Saccharomyces cerevisiae* vineyard populations are important components of the grape/wine system. Besides their direct impact on winemaking, they also constitute an untapped reservoir of genotypes with special technological attributes for the wine industry. Research so far on *S. cerevisiae* populations has focused on spatial distribution on large scales, yet little is known about the genetic variability of populations within viticultural zones and their temporal genotypic variation. Here, *S. cerevisiae* populations from different vineyards in Santorini, a small Aegean island, were genotyped and their genetic diversity was assessed within and between vineyards during two consecutive years. Despite the relative geographical isolation of the island, a relatively high genetic diversity was uncovered. The vast majority of genotypes were vineyard-specific, while in one of the vintages, significant differences in the genotypic composition of vineyards were detected. Overall, higher differences were detected between vintages rather than among vineyards. Notably, only four genotypes were common for the two vintages, three of which were commercial *S. cerevisiae* strains, probably “escapees” from wineries. Nevertheless, the populations of the two vintages were not genetically distinct. Present results highlight the magnitude of genetic diversity in natural wine yeast populations on a small spatial scale, yet the invasion of commercial starters may constitute a potential risk for loss of local yeast biodiversity. However, present results show that industrial strains do not necessarily dominate over the natural strains or their high abundance may be temporary.

## 1. Introduction

*Saccharomyces cerevisiae* has played a major role since ancient times in food bioprocessing, particularly in the production of alcoholic beverages and baking [1]. It most likely originated in China, which harbors the highest genetic diversity in the world [2]. Throughout the centuries, the dispersal of fermentation technology in foods and beverages endorsed yeast selection and multiple domestication events. At the same time, human migration and global transportation activities have relaxed biogeographical barriers and accelerated geographical dispersal. Both the environment and historical aspects have shaped its biogeography. Nowadays, it is widely recognized that the ecology of *S. cerevisiae* is tightly linked to the human history of migration and civilization [3,4,5].

The ecological, genetic, and phenotypic diversity of the species has been the subject of several studies in various biotopes, including vineyards in wine-producing regions worldwide. Research so far has revealed a high level of genetic polymorphism among natural isolates and a complex population structure, which has been ascribed to both ecological factors and geographical attributes [2,6,7,8]. Despite previous extensive work on the genetic diversity of the species, only a few studies have focused on populations associated with grape fruits or ferments [9].

Accumulating data suggest that the grape-associated yeast community may significantly diversify according to different factors, including the sanitary state of grape berries, the climatic conditions, and the farming system [10,11,12,13,14]. Metagenomic analysis of grape must samples revealed that the microbial biogeography of yeast species across California was non-randomly associated with regional, varietal, and climatic factors [15]. The respective biogeographical studies considering the intraspecies spatio-temporal distribution of natural wine yeast populations though are quite limited. Little research so far has pointed to regional differences in natural vineyard populations [16]. Schuller and Casal [17] showed significant differences among *S. cerevisiae* populations isolated from three vineyards (<100 km distance) in Portugal, and more recently, Gayevskiy and Goddard [9] showed the existence of differentiation between natural *S. cerevisiae* populations over a distance of hundreds of kilometers in New Zealand.

The existence of wine yeast biogeographical patterns is of importance to the wine industry since it may connect the wine character of a particular region with the local yeast biota, a so-called “microbial *terroir*” effect. In order to assign a microbial aspect to *terroir*, a temporal stability for regional strains is essential. However, the extent of year-to-year fluctuations in the genotypic variation and the persistence of signature-regional strains in a given area have hardly been examined. Notably, in a survey conducted in Franciacorta and Oltrepo Pavese, Italy, only two strains were shared between different vintages, while not a single strain was recovered from the same vineyard [18]. Here, we assessed the spatio-temporal variation in the vineyard-associated *S. cerevisiae* population of Santorini island, and asked about the degree of invasion of the widely used commercial yeast starters in the autochthonous population.

## 2. Materials and Methods

### 2.1. Grape Samples and Yeast Isolation

**Figure 1:**
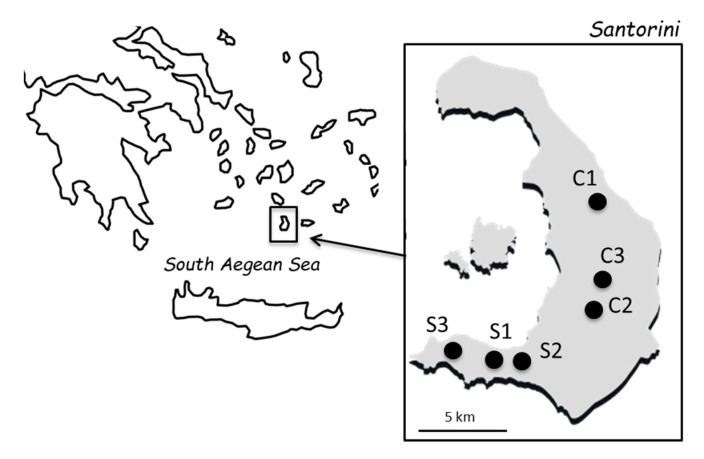
A map showing the location of the six vineyards surveyed in Santorini. The C1–C3 and S1–S3 vineyards are located in the central and southern parts of the island, respectively.

Mature grape samples from the local white grapevine variety “Assyrtiko” (*Vitis vinifera* L.) were collected from 6 vineyards in Santorini, a small island of approximately 73 km^2^ in the southern Aegean Sea, Greece. The vineyards were located in central (C1–C3) and southern (S1–S3) parts of the island (Figure 1). The mean distance between vineyards was 4.6 ± 0.6 km. Sampling was conducted in two consecutive vintages. For each vineyard, 2 to 3 distantly located sampling points were surveyed, avoiding the edges of the field. Each sample consisted of 4–5 kg mature grape bunches collected from a sampling point. Samples were placed into sterile plastic bags and transferred at 4 °C to the laboratory. Grape berries were crushed with a stomacher and ca. 750 mL juice (must) was let to ferment spontaneously in 1000 mL sterile bottles at 20 °C. In total, 30 spontaneous fermentations were conducted. The sugar content, total acidity and pH of the musts were determined just after crushing, according to methods proposed by the International Organisation of Vine and Wine (OIV) in the “Compendium of International Methods of Analysis of Musts and Wines” [19]. Fermentation progress was followed daily by weight determinations. Samples were taken at the end of the alcoholic fermentation (constant weight for two consecutive measurements), diluted (10^−1^ to 10^−6^) in Ringer’s solution, and spread on plates containing WL nutrient agar medium (Oxoid Ltd., Basingstoke, UK). After incubation at 28 °C for 2 days, 16 to 32 colonies showing *S. cerevisiae* morphology from each sample were selected and stored at −80 °C. Species identification was carried out by restriction enzyme analysis of the 5.8S-ITS rDNA region [20], essentially as previously described [21].

### 2.2. Molecular Typing of Isolates

*S. cerevisiae* isolates were differentiated at the strain level by the interdelta region analysis, as described by Legras and Karst [22], using the primer set delta 12 (5′–TCAACAATGGAATCCCAAC-3′) and delta 21 (5′-CATCTTAACACCGTATATGA-3′). Sixteen commercial *S. cerevisiae* starters, including strains that are frequently applied in this area, were also analyzed (Supplementary Table S1). Amplification of genomic sequences flanked by delta elements of retrotransposons TY1 and TY2 was carried out by PCR in a final volume of 25 μL, containing 20 ng of DNA, 1x Buffer Kapa A, 2.5 mM MgCl_2_, 2× BSA, 25 pmol of each primer, 0.2 mM of each dNTP and 1 U of Taq DNA polymerase (KAPA Biosystems, Woburn, Massachusetts, USA). Amplification was performed in a Bio-Rad thermal cycler (T100TM Thermal Cycler, Bio-RAD Laboratories, Emeryville, CA, USA) under the following conditions: initial denaturation at 94 °C for 3 min, followed by 35 cycles of denaturation at 94 °C for 30 s, annealing at 46 °C for 30 s, and extension at 72 °C for 90 s, before a final extension at 72 °C for 10 min. The reaction products were separated on 2% agarose gel and visualized by UV light (GelDoc system, Bio-RAD Laboratories, Emeryville, CA, USA) using a 100 bp DNA ladder (New England Biolabs, Inc., Ipswich, MA, USA) as a molecular size standard.

### 2.3. Data Analysis

For approximate biodiversity estimation, the ratio between the distinct molecular fingerprinting patterns (genotypes) and the number of *S. cerevisiae* isolates was calculated [23,24]. Comparison of proportions was made by the “N-1” Chi-squared test, as recommended by Campbell [25] and Richardson [26]. The dendrogram was constructed by Unweighted Pair Group Method with Arithmatic Mean (UPGMA) clustering, based on the Dice correlation coefficient using PAST software [27]. Differences in the genotype composition among vineyards and between vintages were tested with analysis of similarities (ANOSIM) using PAST [27]. The partitioning of genetic variation with and among vineyards was shown by analysis of molecular variance (AMOVA) using GENALEX 6 [28].

## 3. Results

### 3.1. Fermentations and Genotyping

Six vineyards in Santorini (Figure 1) were surveyed to study the diversity and spatial distribution of vineyard-associated *S. cerevisiae* during two consecutive vintages (Vintage I and II). Grape samples belonged to ‘Assyrtiko’, an indigenous grape cultivar widely cultivated throughout the island. The sugar content of grape musts ranged from 171.5 to 246.4 g/L (mean 212.1 ± 20.6 g/L) in Vintage I, and from 126.2 to 227.7 g/L (179.3 ± 30.4 g/L) in Vintage II. Total acidity, as expressed in g/L tartaric acid, ranged from 6.3 to 7.9 g/L (7.1 ± 0.5 g/L) and from 4.9 to 7.1 g/L (5.9 ± 0.7 g/L) in musts from Vintage I and Vintage II, respectively. Notably, all musts were fermented up to sugar depletion. The amount of CO_2_ released ranged from 92.8 to 128.6 g/L (111.3 ± 9.4 g/L) in Vintage I, and from 87.1 to 119.6 (103.8 ± 11.1 g/L) in Vintage II. The duration of the lag phase ranged from one to four days. *S. cerevisiae* counts reached standard levels at the end of the fermentation, in the range of 7.36 to 8.33 log CFU/mL and from 6.29 to 7.32 log CFU/mL in samples from Vintage I and Vintage II, respectively. Higher yeast counts were observed in Vintage I than in Vintage II (*F* = 8.5, *p* < 0.01). Non-*Saccharomyces* counts were significantly lower than *S. cerevisiae* counts, ranging between 2.76 to 7.16 log CFU/mL and 2.95 to 6.43 log CFU/mL in samples from Vintage I and Vintage II, respectively. In total, 599 *S. cerevisiae* isolates were identified and subjected to genotyping by interdelta sequence analysis, yielding 72 different banding patterns, hereafter referred to as genotypes (Supplementary Figure S1).

### 3.2. Biodiversity and Genotype Distribution

**Figure 2:**
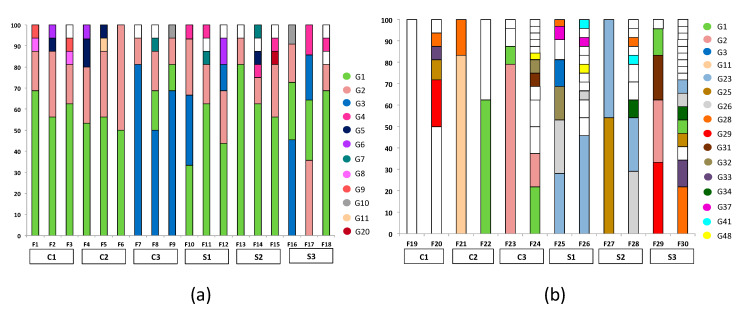
Occurrence of genotypes (%) in different samples (F1–F18) from vineyards (C1–C3 and S1–S3) in (**a**) Vintage I; (**b**) Vintage II. Different colors correspond to distinct genotypes isolated from at least two vineyards or vintages, while white indicates genotypes isolated from a single vineyard in a given vintage.

In Vintage I, 22 distinct genotypes were identified out of 279 *S. cerevisiae* isolates analyzed (7.9% genetic biodiversity). Two to six genotypes were isolated at the end of each fermentation course (Figure 2a), while each vineyard harbored 5 to 9 different genotypes (average 7.2 ± 1.3). Comparison of yeast molecular patterns with those of commercial starters revealed that the genotypes G1, G2 and G3 shared identical banding profiles with those of the commercial strains Level2 TD kit, Lalvin QA23, and Uvaferm 228, respectively. The majority of the genotypes (63.6%) were vineyard-specific, i.e., associated with a single vineyard (Figure 2a, Table 1). The commercial strains G1 and G2 were highly dispersed across the island, associated with all vineyards and frequently isolated from different ferments (samples). G2, in particular, was the most abundant genotype, found in all samples, followed by G1. ANOSIM revealed significant differences in the genotypic composition of vineyards (*R* = 0.315, *p* < 0.01).

In Vintage II, 320 isolates were assigned into 54 distinct genotypes. The total percentage of biodiversity was 17%, significantly higher than the one estimated previously (*p* < 0.001). The number of different genotypes per sample ranged substantially (from 1 up to 17), being much higher than the previous year (Figure 2b). Each vineyard harbored 4 to 21 genotypes (average 12.3 ± 6.3). Similar to Vintage I, the majority of genotypes (75.9%) was vineyard-specific (Figure 2b, Table 1). G23 was the most frequently encountered genotype in different ferments, while G28 was the most abundant genotype among vineyards (83% of the vineyards). Notably, only four genotypes were common for the two vintages, and these corresponded to the commercial strains G1, G2, and G3, along with the autochthonous G11. The commercial strains were encountered at rather low percentages (<10%), with G11 being recorded from only 6.3% of the isolates (Figure 2b). As opposed to Vintage I, the genotypic composition of vineyard-associated groups did not differ significantly among each other, as revealed by ANOSIM (*R* = 0.197, *p* = 0.191). A two-way crossed ANOSIM analysis showed a much higher effect for the vintage (*R* = 0.743, *p* < 0.001), rather than for different vineyards (*R* = 0.256, *p* = 0.01) on the genotypic composition of vineyard groups.

### 3.3. Genetic Similarities of Genotypes

**Figure 3:**
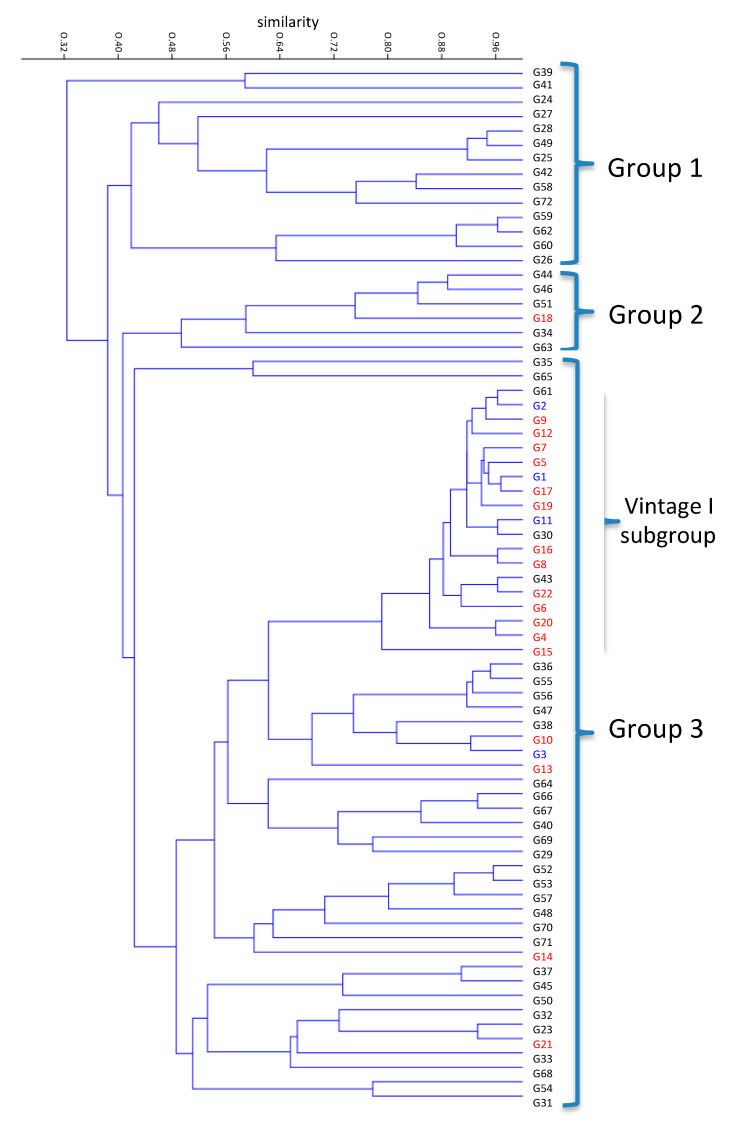
Unweighted Pair Group Method with Arithmatic Mean (UPGMA) clustering of genotypes. Red and black letters correspond to genotypes of Vintage I and Vintage II, respectively. Genotypes common to both vintages are indicated in blue.

UPGMA clustering of different genotypes isolated from both vintages revealed three main groups (Figure 3). Both Group 1 and Group 2 were almost solely composed of genotypes isolated in Vintage II. Group 3 was more diverse, including genotypes from both vintages and also the commercial strains G1, G2, and G3. An interesting clustering of the Vintage I’s genotypes in a tight subgroup (Vintage I subgroup) within Group 3 was observed. The genotypes of the “Vintage I subgroup” showed relatively high genetic similarity to each other and to the common genotypes between the two vintages. Nevertheless, an AMOVA test did not show significant genetic differentiation between the genotypes of the two vintages (*p* = 0.544) (Table 2).

## 4. Discussion

Vineyard indigenous microbiota has attracted renewed vigor lately due to the increasing demand for wines produced through natural fermentations or by autochthonous yeasts. Differences in the composition and spatial distribution of microbial communities or populations have been described on large scales (over 100 km), but little is known about the respective patterns on small scales [29]. Furthermore, besides spatial scales, the temporal variation of yeast vineyard populations has been scarcely investigated. However, year-to-year variation in the composition of yeast populations and the potential persistence of certain regional signature genotypes are important components of the so-called microbial *terroir* concept [30].

Here, the spatio-temporal genetic diversity patterns of *S. cerevisiae* populations in a geographically isolated island were evaluated. The observed genotypic richness recorded was variable, ranging from 1 to 17 genotypes in different ferments. These values are generally within the range of previous studies, i.e., 1 up to 20 genotypes per ferment [13,24,31]. Both the genotypic richness and the percentage of biodiversity were significantly higher in Vintage II. A temporal variation was also observed in the composition of vineyard genotypic groups. Although ANOSIM revealed significant differences among vineyards in Vintage I, no respective differences were detected in Vintage II. A two-way ANOSIM showed that the variation in the genotypic composition of vineyard-associated groups was higher between vintages rather than among vineyards. Relevant information on the evolution of *S. cerevisiae* populations over time in a given area is very limited [29,32]. In a previous study, it was shown that the level of differentiation of vineyard-associated *S. cerevisiae* populations during consecutive years was similar to that observed among different vineyards within the same year [17]. In line with our results, *S. cerevisiae* populations of different vineyards and cellars in two Italian vine-growing regions differed significantly between vintages, rather than among geographical sites [18]. A high degree of annual discrepancy in the persistence of vineyard-associated genotypes seems also to apply in wine cellars [18], except for a few dominant strains that are repeatedly found over the years [33]. This may not be surprising considering the wide use of commercial starters in wineries and their rather diminishing effect on the diversity of indigenous *S. cerevisiae* populations [34]. It has been suggested that, as the harvest period finishes, wine yeasts may hibernate in nutrient-deficient niches of vineyards or in the guts of insects [15,35,36]. Under such conditions, yeasts are not supposed to multiply clonally, but rather follow sexual reproductive cycles [36]. This possibility, combined with the annual variation in weather conditions, may explain the differentiation of the genetic background in vineyard-associated wine yeasts over time.

Notably, only four common strains could be recovered in both vintages, with three of them sharing the same molecular patterns with commercial yeast starter strains commonly used in the local wineries. The extensive use of starter cultures in winemaking is currently emerging as a critical factor that affects natural biodiversity in viticultural regions [31]. Commercial strains are used to conduct the fermentation in wineries and are actively transferred to the field through liquid and solid winery deposits. As a consequence, the natural biodiversity of *S. cerevisiae* in vineyards could be distorted by the dispersal of starters. It has been previously shown that the release of starters was confined only to a few meters around the winery for short periods of time [37]. In contrast, in a recent study, an extensive commercial yeast diaspora was observed in large winemaking areas [31]. These authors also detected that the commercial starters were spread permanently in the vineyard ecosystem, suppressing the native yeast biodiversity. Observations similar to the one above were made in this study for Vintage I, where two starter strains were highly abundant in all ferments. Several genotypes that were genetically related to the commercial strains above were also isolated in the same vintage, in accordance with previous observations [31]. However, commercial strain evenness was reversed during the next harvesting season. The widespread commercial strains G1 and G2 from Vintage I were also present in Vintage II, but at much lower incidences. Interestingly, yeast biodiversity in Vintage II was significantly higher than that observed previously [31], and the most abundant genotype recovered (83% of the vineyards) was an autochthonous strain.

Taken together, present data corroborate previous results proposing the extensive use of yeast wine starters as a risk for loss of genetic diversity of autochthonous yeast populations [31,38], but also show that the industrial wine strains may not necessarily dominate over the natural strains, and importantly, their abundance seems to be temporary. Present results may add to our knowledge for elucidating patterns of *S. cerevisiae* spatio-temporal differentiation on a small scale. Besides, the establishment of *S. cerevisiae* strain collection from Santorini, an island with a millennia history in winemaking, is of great importance for preserving regional genetic resources for the sustainability of the wine and food industry.

## Figures and Tables

**Table 1:** Occurrence of genotypes in different vineyards (%). Common genotypes in both vintages are highlighted by various colors.

Vintage I		Vintage II	
Genotype Code	Vineyards (%)	Genotype Code	Vineyards (%)
**G2**	100	G28	83.3
**G1**	100	**G1**, G23	50
**G3**	50	G26	50
G4	50	G25	50
G5	50	**G2**	33.3
G6, G7	50	G29, G31–G34, G41, G48	33.3
G10	33.3	G37	16.7
G8–G9	16.7	**G3**, **G11**, G24, G27, G30, G35–G36, G38–G40, G42–G47, G49–G72	16.7
**G11**-G22	16.7		

**Table 2:** Results of the analysis of molecular variance (AMOVA) showing the partitioning of genetic variation between and within populations of the two vintages.

Source of Variation	d*f*	Variance Components	Variation (%)	PhiPT	*p*-Value
Between populations	1	0.151	0	−0.013	0.544
Within populations	75	19.018	100		

## Supplementary Materials

The following are available online at https://www.mdpi.com/2304-8158/9/5/561/s1, Table S1: Set of 16 *S. cerevisiae* commercial strains included in the analysis. Figure S1: Interdelta banding patterns of Vintage I strains (**A**) and representative strains of Vintage II (**B**).
