# Supplementary material for: Patterns of Genetic Diversity and the Invasion of Commercial Starters in Saccharomyces cerevisiae Vineyard Populations of Santorini Island
Source: Foods. 2020 May 2;9(5):561. doi: 10.3390/foods9050561 (PMC7278685; doi:10.3390/foods9050561)
Supplement: Supplementary file 1 [file foods-09-00561-s001.pdf]

Table S1. Set of 16 *S. cerevisiae* commercial strains included in the analysis.

Product Name	Supplier
Actiflore BO 123	Laffort
Actiflore F33	Laffort
Cross Evolution	Lallemand
Fermicru 4F9	Oenobrands
Fervens berry	Dal Cin
Lalvin 71B	Lallemand
Lalvin EC-1118	Lallemand
Lalvin QA23	Lallemand
Level2 TD	Lallemand
Uvaferm 228	Lallemand
Viniflora Rhythm	Christian Hansen
Vitilevure B+C	Martin Vialatte
Vitilevure Elixir	Martin Vialatte
Zymaflore FX10	Laffort
Zymaflore Spark	Laffort
Zymaflore X5	Laffort

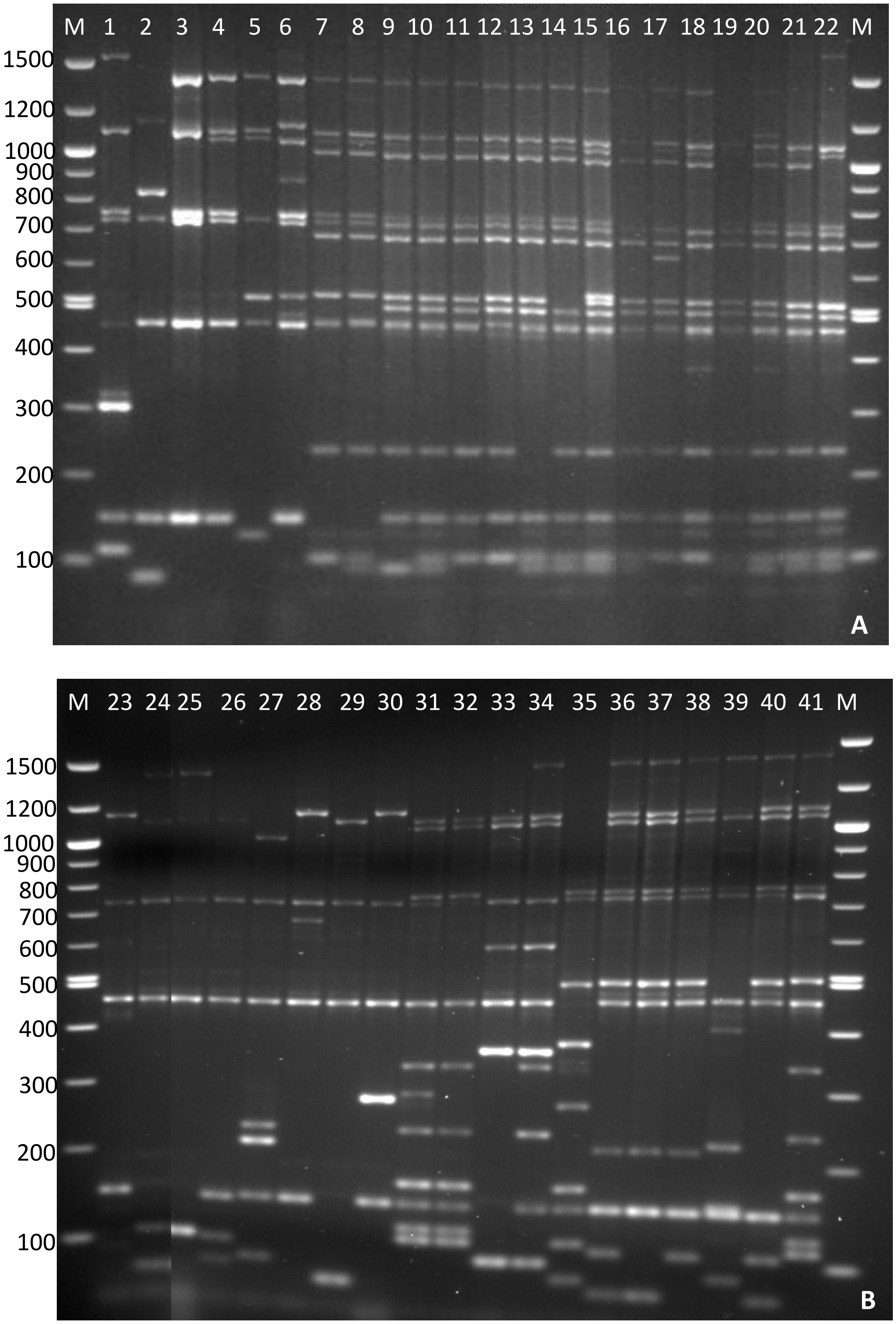

Figure S1. Interdelta banding patterns of Vintage I strains (**A**) and representative strains of Vintage II (**B**). Lane 1, G14; 2, G18; 3, G10; 4, G3; 5, G21; 6, G13; 7, G20; 8, G4; 9, G5; 10, G1; 11, G2; 12, G12; 13, G9; 14, G7; 15, G17; 16, G22; 17, G16; 18, G8; 19, G15; 20, G6; 21, G19; 22, G11; 23, G44; 24, G37; 25, G45; 26, G34; 27, G63; 28, G46; 29, G50; 30, G51; 31, G52; 32, G53; 33, G54; 34, G31; 35, G26; 36, G36; 37, G55; 38, G56; 39, G29; 40, G47; 41, G57. M, 100 bp DNA ladder (numbers in bp).
